# First Report of OvoA Gene in Marine Arthropods: A New Candidate Stress Biomarker in Copepods

**DOI:** 10.3390/md19110647

**Published:** 2021-11-20

**Authors:** Vittoria Roncalli, Chiara Lauritano, Ylenia Carotenuto

**Affiliations:** 1 Integrative Marine Ecology Department, Stazione Zoologica Anton Dohrn, Villa Comunale, 80121 Napoli, Italy; ylenia.carotenuto@szn.it; 2 Marine Biotechnology Department, Stazione Zoologica Anton Dohrn, Villa Comunale, 80121 Napoli, Italy; chiara.lauritano@szn.it

**Keywords:** zooplankton, natural products, antioxidant, transcriptome mining

## Abstract

Ovothiol is one of the most powerful antioxidants acting in marine organisms as a defense against oxidative stress during development and in response to environmental cues. The gene involved in the ovothiol biosynthesis, OvoA, is found in almost all metazoans, but open questions existed on its presence among arthropods. Here, using an in silico workflow, we report a single OvoA gene in marine arthropods including copepods, decapods, and amphipods. Phylogenetic analyses indicated that OvoA from marine arthropods separated from the other marine phyla (e.g., Porifera, Mollusca) and divided into two separate branches, suggesting a possible divergence through evolution. In the copepod *Calanus finmarchicus*, we suggest that OvoA has a defense role in oxidative stress as shown by its high expression in response to a toxic diet and during the copepodite stage, a developmental stage that includes significant morphological changes. Overall, the results of our study open possibilities for the use of OvoA as a biomarker of stress in copepods and possibly also for other marine holozooplankters. The finding of OvoA in copepods is also promising for the drug discovery field, suggesting the possibility of using copepods as a new source of bioactive compounds to be tested in the marine biotechnological sector.

## 1. Introduction

Ovothiols are low molecular weight thiol-containing methylated amino acids with unique antioxidant properties that are broadly distributed among invertebrates, microalgae, protozoans, and bacteria [1]. Playing a key role in the maintenance of cellular redox homeostasis, ovothiols allow the organism to overcome environmental stress conditions. In marine organisms, ovothiols play a key role also during development as suggested by their antioxidant activity during oxidative stress at fertilization and larval development in the sea urchin [2], and during gametogenesis in the mollusc *Mytilus galloprovincialis* collected from polluted sites [3]. Ovothiols also act as a defense against the immune system of host cells during parasite infections [4,5], and as a protective compound in the mucus of Polychaeta [6]. These molecules have also been suggested as signaling molecules released in the urine of cephalopods [6], in pathways induced by light in microalgae [7,8], and as pheromones in marine worms and cone snails [9].

Recent studies showed new ovothiol bioactivities, highlighting interesting possible applications of this antioxidant in the pharmaceutical sector. Ovothiol A isolated from the sea urchin *Paracentrotus lividus* oocytes, reduced the cell viability of the human liver carcinoma cell line (Hep-G2) by activating autophagy [10]. Additional possible ovothiol A antiatherogenic activities have been found by cell-based assays suggesting its application for cardiovascular diseases associated with oxidative and inflammatory stress, as well as endothelial dysfunction [11]. In addition, in an in vivo study in mice ovothiol A showed activity against liver fibrosis progression [12].

**Figure 1:**
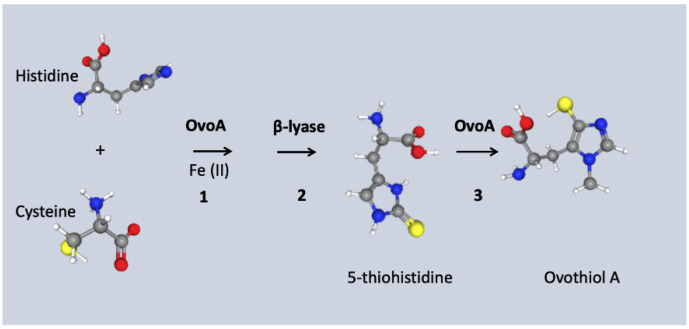
Ovothiol A biosynthetic pathway. Schematic representation of Ovothiol A pathway which consists of three steps (1–3) catalyzed by two enzymes (in bold). (1) OvoA enzyme (5-histidylcysteine sulfoxide synthase) catalyzes the addition of the cysteine sulfur group into histidine to produce an intermediate (not shown); (2) The intermediate (not shown) is cleaved by sulfoxide **β** lyase (OvoB) into thiohistidine which is then (3) methylated by OvoA to ovothiol (π-N-methyl-5-thiohistidine). Chemical structures were downloaded from the National Center for Biotechnology Information (NCBI) PubChem database. Histidine: PubChem Identifier CID: 6274, https://pubchem.ncbi.nlm.nih.gov/compound/Histidine, accessed on the 3 November 2021; Cysteine: PubChem Identifier CID: 6419722, https://pubchem.ncbi.nlm.nih.gov/compound/Cysteine, accessed on the 3 November 2021; OvothiolA: PubChem Identifier CID: 130131 https://pubchem.ncbi.nlm.nih.gov/compound/Ovothiol-A, accessed on the 3 November 2021.

The ovothiol biosynthetic pathway includes three enzymatic steps in which OvoA is the key enzyme with a bifunctional role. First, the OvoA enzyme, 5-histidylcysteine sulfoxide synthase, catalyzes the addition of the cysteine sulfur group into histidine to produce an intermediate; subsequently the intermediate is cleaved by sulfoxide b-lyase (OvoB) into thiohistidine that is finally methylated to ovothiol (π-N-methyl-5-thiohistidine) by OvoA. This final step is specific of the S-adenosylmethionine (SAM) methyltransferase domain situated in the C-terminal of the enzyme. OvoA also contains an N-terminal DNA damage-inducible (DinB) superfamily domain and a formylglycine-generating sulfatase (FGE-sulfatase) domain that contains the recognition/binding sites for the substrates (cysteine and histidine) [13] (Figure 1). The methyltransferases that can methylate the a-amino group of ovothiol A to form ovothiol B and C are not yet known.

Braunshausen and Seebeck [14] were the first to characterize the OvoA gene from the bacterium *Erwinia tasmaniensis* and the protozoan *Trypanosoma cruzi*. They also found homologous OvoA enzymes in more than 80 genomes ranging from proteobacteria to uni- and multicellular eukaryotes [14]. From a phylogenetic point of view, OvoA has been reported in many metazoans including Porifera, Emichordata, and Placozoa. Through evolution, the gene was lost twice, once in the common ancestor of nematodes and arthropods and once in the ancestor of Osteichthyes fish [15]. In freshwater fish, ovothiol A has been identified in the metabolites of the lens and other tissues as well as in the eggs, suggesting that these organisms might not have the gene, but are able to acquire the metabolite through their diet [16,17]. In contrast, still little is known of arthropods; it has been suggested that the lack of OvoA in most terrestrial species (e.g., insects) could be related to a specific role of ovothiol in the transition from the aquatic to the terrestrial environment [1,15]. Recently, Brancaccio and coauthors [18] conducted a genomic and metagenomics data mining to investigate the distribution and diversification of the enzymes involved in ovothiol biosynthesis in bacteria. They observed a horizontal gene transfer event of OvoB from Bacteroidetes living in symbiosis with Hydrozoa and suggested that the evolution of ovothiol biosynthesis may have involved symbiosis processes [18]. Overall, from all these studies, it is clear that ovothiol A is an important antioxidant as it is conserved in many metazoans; however, the studies highlight the need to better investigate the presence of OvoA gene in other phyla.

Gene expression changes of OvoA have been reported in *P. lividus* during development and when exposed to stress conditions. Relative expression of OvoA was high in eggs and decreased immediately after fertilization, remaining low in the early developmental stages (early and the swimming blastula) with a final significant increase in the last larval stage (pluteus) [2]. A significant increase in the expression of OvoA has also been reported in larvae exposed algae and to the metals Cd and Mn [2,19]. Overall, these results suggested that in *P. lividus* ovothiol may act as a protective compound against environmental stressors, and/or as a regulation factor during development.

The aim of this study was to explore the occurrence and diversity of the OvoA gene in marine arthropods. Since the OvoB gene has not been identified in metazoan genomes or transcriptomes, except hydrozoans [1,18], we focused our investigation on OvoA. Using a well-established in silico workflow we mined the new publicly available transcriptomic resources for copepods, expanding the searches also to malacostraca. Copepods are an important component in most trophic marine food webs [20,21,22]. As part of zooplankton, those tiny crustaceans live in highly variable environments and are constantly subject to natural and anthropogenic-related stressful conditions that might compromise their cellular redox homeostasis [23,24,25,26,27]. However, in these herbivorous consumers, still little is known on which genes are activated during detoxification and which genes are responsible of antioxidant production. Given the high antioxidant properties of ovothiol in many marine organisms, we decided to examine the occurrence of OvoA gene in copepods. Using an in silico workflow, we identified OvoA transcripts in copepods but also in other marine arthropods. The identified OvoA transcripts were used in a phylogenetic analysis to support their annotation and to investigate their relationship to other marine metazoans. Lastly, using previous RNA-Seq-based studies, the expression of OvoA across development and after feeding on toxic phytoplankton species was investigated in two crustacean copepods, to evaluate the potential role of ovothiol as protective antioxidant in these holozooplankters.

## 2. Results

### 2.1. Identification of OvoA Encoding Transcripts in Marine Arthropods

**Figure 2:**
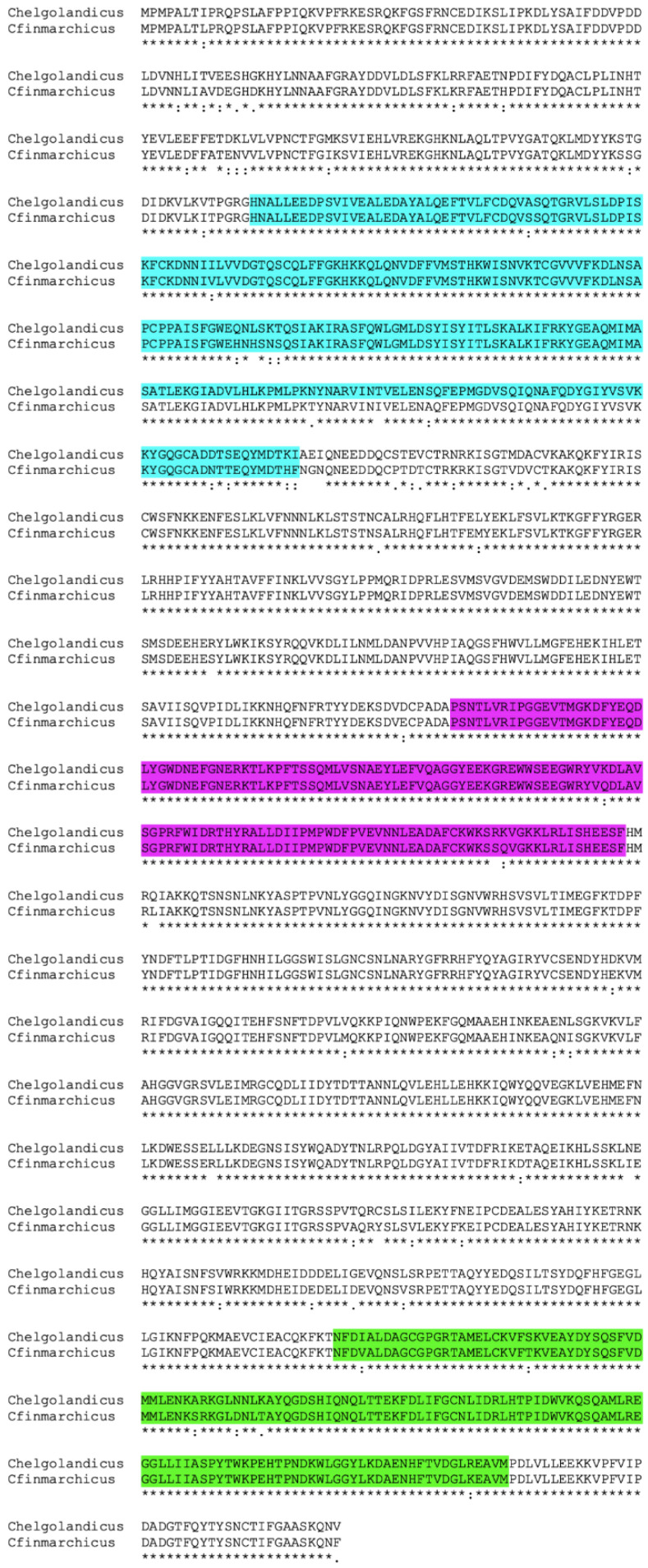
OvoA alignment in the copepods *Calanus finmarchicus* and *C. helgolandicus*. Protein alignment of the deduced OvoA sequenecs for the copepod *C. finmarhcicus* and *C. helgolandius*; “*” beneath the alignment indicates residues that are identical in the two sequences while “:” and “.” indicate conservatively substituted aminoacids shared between the protein pair. The three strunctural functional domains identified by the Pfam database are highlighted as follows: DNA damage-inducible (DinB) (light blue), formylglycine-generating sulfatase (FGE-sulfatase) (magenta), and S-adenosylmethionine (SAM) methyltransferase (green).

In this study we report for the first time that marine arthropods also possess OvoA, a key player gene of ovothiol biosynthesis. By mining the publicly available transcriptome database (TSA) limiting to Arthropoda, we identified a single transcript encoding OvoA in 19 copepods and nine malacostracans (Table 1, Table S1). Within the copepod subphylum, OvoA was identified in 11 Calanoids, three Cyclopoids, three Harpacticoids and two Siphonostomatoids. The Calanoida order, with the highest number, included mostly individuals from the Calanidae family such as *Calanus finmarchicus*, *C. helgolandicus* and *Neocalanus flemingeri*. Among the malacrustracans, OvoA were found in seven decapods and two amphipods (e.g., *Gammarus pulex*, *G. fossarum*) (Table 1, Table S1). Reciprocal blast confirmed that all transcripts were annotated as protein OvoA with 70% encoding for full length proteins. Structural domain analysis confirmed the presence of the three expected functional domains, the DNA damage-inducible (DinB), the formylglycine-generating sulfatase (FGE-sulfatase), and the S-adenosylmethionine (SAM) methyltransferase) domains, as shown in the copepods *C. finmarchicus* and *C. helgolandicus* (Figure 2).

### 2.2. Phylogenetic Analysis of OvoA Transcripts from Marine Metazoans

**Figure 3:**
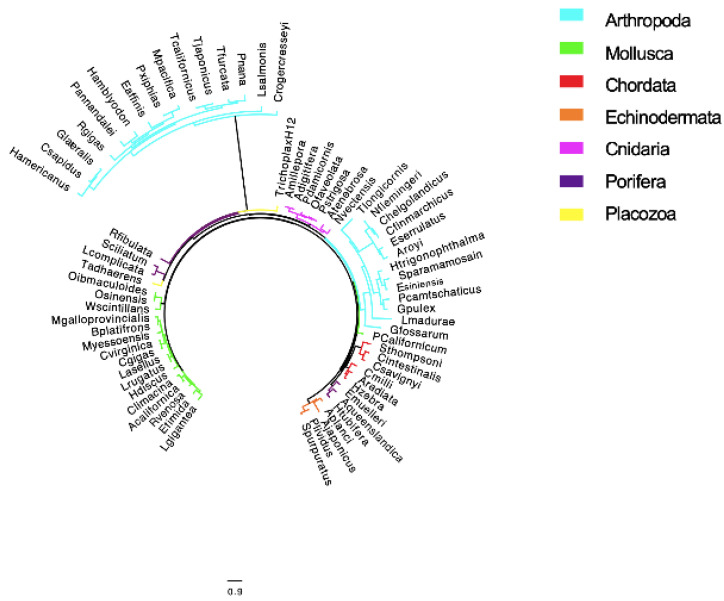
Phylogenetic tree for OvoA gene in marine metazoans. The unrooted tree shows the relationships between OvoA identified in this study for marine arthropods and OvoA from selected marine metazoans. Amino acid sequences were aligned using ClustalW, and FASTTREE was used to build a maximum-likelihood phylogenetic tree (Galaxy v. 2.1.10+ galaxy1) using the protein evolution model JTT+ CAT. Color coding refers to the different phylum: light blue = Arthropoda (sequences identified in this study), green = Mollusca, red = Chordata, orange = Echinodermata, pink = Cnidaria, purple = Porifera, yellow = Placozoa. Scale bar indicates the number of amino acid substitution per site.

Phylogenetic analysis of OvoA sequences deduced in this study for marine arthropods was used to support their annotation and to investigate their relationship to each other and to those from other marine metazoans. The analysis generated a consensus tree with several clades representing the different phyla. Consistent with what has been previously reported for marine metazoans, OvoA sequences from Mollusca, Echinodermata, Cnidaria, and Chordata phylum cluster separately and in individual clades (Figure 3). The OvoA sequences identified in this study for marine arthropods separated into two clades (Figure 3). The first clade, highly separated from all other metazoans, is closely related to OvoA from Placozoa and from two Porifera (Figure 3). This clade included 15 OvoA sequences, including 12 sequences from copepods and three from decapods (Figure 3). The second clade, with a total of 13 OvoA sequences, included seven copepods, four decapods and two amphipods. The seven copepods included five OvoA sequences from Calanoida and two sequences from Cyclopoida (Figure 3).

### 2.3. Ovo A Expression in Calanus finmarchicus and C. helgolandicus

The expression of OvoA was examined in the copepods *C. finmarchicus* and *C. helgolandicus* using previous RNA-Seq data for these copepods feeding on toxic algal species [28,29].

**Figure 4:**
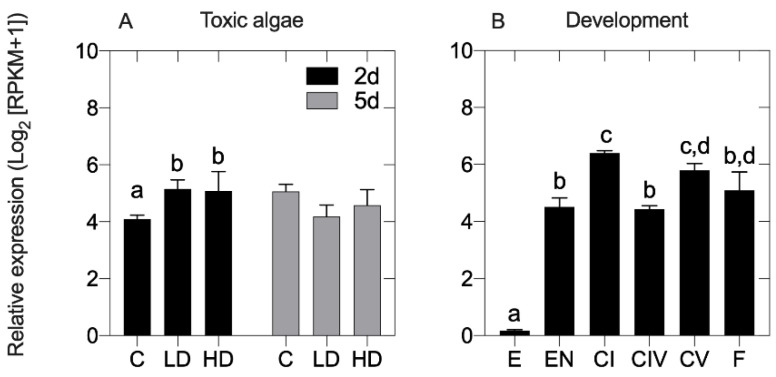
OvoA expression in *Calanus finmarchicus*. In both graphs, relative expression was normalized by length RPKM (Log2) adding pseudocounts of 1. (**A**) Expression of OvoA in adult females exposed for 2 days (2d) and 5 days (5d) to a control diet *Rhodomonas* sp. (C), and low (LD) and high doses (HD) of the toxic dinoflagellate *Alexandrium fundyense* [29]. Bar graphs indicate mean with standard deviation (SD) of the three replicates in each diet; (**B**) Expression of OvoA across development: embryos [E], early nauplii (NII-NIII) [EN], early copepodites [CI], late copepodites (preadult CIV and CV) [CIV] [CV], females [F]. Bar graphs indicate 2-way ANOVA of the three replicates in each sample (exception CI and CIV with two replicates). Significant differences (*p* < 0.05; 2-way ANOVA followed by post-hoc Tukey’s test) among stages are indicated by small letters over the bars.

In *C. finmarchicus*, exposure to the saxitoxin producing dinoflagellate *Alexandrium fundyense* induced changes in the expression of OvoA (Figure 4A). The increase in expression of OvoA did not depend on the dose of the toxic algae but was time affected (Figure 4A). Specifically, at two days, OvoA was found up-regulated in females fed with toxic algae at low (LD) and high (HD) doses (LD = 5.1 ± 0.11 Log2 [RPKM + 1], HD = 5.03 ± 0.5 Log2 [RPKM + 1]) compared with females on the control diet *Rhodomonas* sp. (Figure 4A). In contrast, a longer exposure to the dinoflagellate had no significant effect; at five days, the expression of OvoA was similar between diets and not significantly different compared with females on the control diet (Figure 4A).

In *C. helgolandicus* exposure to a toxic diet did not affect the expression of OvoA. In females exposed for 5 days to the toxic diatom *Skeletonema marinoi*, OvoA expression was not significantly different from the expression of females feeding on the control diet *P. minimum* (TOXIC = 9.97 ± 1.35, Log2 [RPKM + 1]; CONTROL = 9.80 ± 0.36, Log2 [RPKM + 1]). The reported lack of OvoA differential expression after 5 days on the toxic algae is similar to that reported for *C. finmarchicus* (Figure 4A).

Changes in the expression of OvoA were also examined in *C. finmarchicus* through development. Using previously published RNA-Seq expression data, we examined the expression of OvoA in different stages including embryos (E), early nauplii (EN), early copepodites (CI), late copepodites (CIV and CV), and adult females (F). Compared with all other stages, the lowest and significantly different expression of OvoA was found in embryos (0.2 Log2 [RPKM + 1]) (Figure 4B). The OvoA expression significantly increased across development reaching its peak in the first copepodite stage (CI) (6.2 ± 0.19 Log2 [RPKM + 1]). A high expression was also maintained through the pre-adult stage (CV) (5.7 ± 0.19 Log2 [RPKM + 1]) but it decreased in the adult stage (Figure 4B).

## 3. Discussion

Ovothiols are small sulfur-containing natural metabolites playing a key role in protecting the organisms against oxidative stress. For its chemical properties and low molecular weight, Ovothiol A is one of the strongest antioxidants reacting with ROS and radicals significantly faster than other natural thiols. Ovothiols are biosynthesized in a two-way step with OvoA, 5-histidylcysteine sulfoxide synthase, being the key regulator. The OvoA gene is highly conserved and found in almost all marine metazoans including Porifera, Cnidaria, Hemicordata, and Echinoderamata [15]. Interestingly, some organisms such as insects and fish, are unable to produce those secondary compounds. This seems to be related to the two gene loss event that occurred through evolution for the ancestral Ecdysozoa (nematodes and arthropods) and the ancestor of Osteichthyes fish. Although fish lack the OvoA to biosynthesize the compounds, ovothiols have been found in different tissues of freshwater fish suggesting that those organisms acquire the metabolite through their nutrition [15]. For arthropods, there was no evidence of OvoA gene and no reports on the presence of ovothiols. In our study, we present the results for the mining of the publicly available transcriptomic resources on the National Center for Biotechnology Information (NCBI) database (TSA) limiting the results to the marine arthropods, mostly represented by the Crustacea subphylum. However, during our searches, OvoA hits resulted also for terrestrial species. For the completeness of our mining (data are not shown) we further examined those sequences with reciprocal blast and structural domain analysis as part of our workflow. This resulted in the identification of OvoA transcripts from eight insects (6 Hemiptera and 2 Diptera). Considering that the focus of the study is on marine organisms, we did not expand the investigation on the terrestrial arthropods. However, our preliminary results might suggest that more investigations are needed in order to clarify also the presence of this gene in terrestrial species.

Marine arthropods are among the most distributed living organism in aquatic environment; copepods, the insects of the sea, dominate the zooplankton. Zooplanktonic organisms have a key role in the energy transfer to higher trophic levels. Through their lifecycle, copepods are commonly exposed to abiotic and biotic stressors that disturb their cellular homeostasis with negative effects on their fitness and in extreme conditions their survival. To cope with the stress, organisms typically activate a cellular stress response (CSR) [30]. This response has the goal to repair and prevent macromolecular damage, to activate cell cycle checkpoints, to reallocate energy resources, and in extreme cases to activate a programmed cell death [30]. The critical part of the CSR is the antioxidant system, a set of enzymes acting against the oxidative protein damage induced by elevated concentration of reactive oxygen species (ROS). Antioxidant proteins, commonly used as biomarkers of oxidative stress, include thioredoxin/thioredoxin reductase, glutaredoxin/glutathione/glutathione reductases, metallothioneins, and cytochrome P450 proteins.

The increase of new transcriptomic resources for marine arthropods, mostly for organism from the copepoda order, has provided the opportunity to expand targeted gene discovery in these organisms. A better understanding of the complexity of gene families of interest, in particular those associated with response to stress, opens new opportunities to discover new biomarkers that could be used for functional studies. For its antioxidant properties, OvoA could be a new biomarker to evaluate antioxidant stress responses in marine organisms. Here we focused on the target identification in marine arthropods of the transcripts encoding for the OvoA enzyme. Our study describes the ovothiol gene distribution in many marine arthropods providing the first report of OvoA gene in the Arthropoda phylum. For mining transcriptomic resources for marine organisms, which included 19 copepods and nine malacrostacans, we found in each organism a single OvoA transcript encoding protein. Seventy percent of the proteins deduced from the predicted OvoA transcript appear to be full-length and showed the expected structural hallmark. Phylogenetic analysis was used to support the annotation and also to evaluate the relationship of OvoA from marine arthropods with other marine metazoans. Based on our results we found that the OvoA sequences from marine arthropods are significantly different from the other metazoans by clustering in independent clades. However, to our surprise, the OvoA sequences separated in two clades that included both copepods and malacrostacans with no distinction between copepod orders. The separation of two clades might suggest that through evolution the OvoA has diverged, however more studies are needed.

In many marine organisms, ovothiols are known for their role in the oxidative response against environmental stressors. In sea urchins, ovothiols are significantly over-expressed in response to metals and toxic algae [2]. In the starlet sea anemone *Nematostella vectensis* OvoA has been suggested as protector against environmental pollutants [31]. A significant high expression of OvoA was found in organisms exposed to dispersant and/or sweet crude oil exposure alone or combined with ultraviolet radiation (UV) [31]. Here, to support a possible role of OvoA as antioxidant in copepods, we used previously generated RNA-Seq data for two calanoid copepods exposed to harmful microalgae to examine the expression of OvoA. In the previously published study, the physiological response of *C. finmarchicus* females exposed to the neurotoxin-producing dinoflagellate *Alexandrium fundyense* was investigated after two- and five-days exposure [28]. The authors reported that at 2 days, *C. finmarchicus* activates a cellular stress response that involves differential expression of many genes associated with molecular chaperoning, apoptosis, cell cycle checkpoint, intracellular signaling, and protein turnover [28]. Although detoxification was not the major component of the response, up-regulated with the diet there were also some antioxidant biomarkers such as glutathione S-transferase (GST), sulfotransferase, and thioredoxin. Here, we found that the expression of OvoA was significantly high in *C. finmarchicus* exposed to the toxic diet compared with individuals on a control diet. The differential regulation of OvoA at 2 days reported here, is consistent with a role of OvoA as antioxidant as part of the CSR previously reported [28]. Furthermore, the lack of significant difference in the expression between the two toxic diets (low and high doses) agrees with the findings that the *C. finmarchicus* response to the toxic algae is not dose dependent. At 5 days, we did not report differences in the expression of OvoA between individuals on the toxic diets (both doses) and the control. This agrees with the fact that at 5 days the *C. finmarchicus* CSR became a cellular homeostatic response, characterized by fewer differentially expressed genes [28]. A homeostatic response is activated when the organism physiologically adapts to new conditions and starts to re-establish homeostasis by counteracting the stress in a specific way [30].

Consistent with *C. finmarchicus*, we also observed no significant differences in OvoA expression when the congener *C. helgolandicus* was fed for 5 days on the oxylipin-producing diatom *S. marinoi*, with respect to the control diet. Oxylipins are lipid-derived info chemicals that regulate the structure and functioning of natural phytoplankton communities [25], and also act as defensive compounds against consumer copepods, by inducing offspring abnormalities, thereby reducing population recruitment at sea [25,32]. Interestingly, the same *C. helgolandicus* females for which we examined the expression of OvoA, showed strong up regulation of genes involved in stress response and xenobiotic detoxification, such as GST and cytochrome P450 [29]. Hence, it is possible that these *C. helgolandicus* individuals were activating a detoxification system based on other antioxidants than ovothiols, that protected the adult copepod from the direct ingestion of the harmful diet. Although we did not have information on OvoA expression in *C. helgolandicus* females feeding for 2 days on *S. marinoi*, a previous study showed that these copepods activated a CSR by over-expressing genes encoding for cellular chaperons, as well as for proteins involved in signal transduction pathways and cell cycle [33]. Given the similar cellular response of the two copepod species when exposed for short feeding times to harmful algal diets, we could speculate that ovothiols may play a role in the antioxidant defensive system of *C. helgolandicus*, as well. However, further studies are needed to confirm this hypothesis.

In addition to their role in response to stressor, ovothiols play a role also during development [2]. In the sea urchin *P. lividus*, a significant high expression of the OvoA gene was found not only in the post-fertilized eggs but also in the pluteus stage during larval development. At the pluteus stage, the organism undergoes a series of morphological changes leading to the final metamorphosis. Alteration of the redox homeostasis is common through development as a consequence of oxygen radicals or reactive oxygen species (ROS) that act as primary or secondary messengers to promote cell growth [34]. The periods associated with morphological changes are characterized by an increase of metabolic activity and apoptosis that leads to elevated concentration of ROS. Thus, it is not strange that OvoA transcript is highly expressed in those stages of development due to its role in ROS scavenging. Copepods have a molt cycle with six naupliar and five copepodite stages [35]. A significant high expression of OvoA was found here in *C. finmarchicus* in the first and the last copepodite stages. Those two stages are key through development. Between the six naupliar and the first copepodite stage the organism undergoes morphological rearrangements while the 5th copepodite stage is when the organism sexually differentiates [35]. Although copepods have a molt cycle that does not involve a pupal stage as in sea urchin (with final metamorphosis), the high expression at those two key stages is like the sea urchin findings, with OvoA playing a key role during late development. Taken together, the pattern of expression reported for OvoA transcript in *C. finmarchicus* fed on the toxic diet suggests that the production of ovothiols is a possible adaptive strategy to cope with environmental stressors. A differential expression through development could be associated either with an antioxidant response to the byproducts that are generated during metamorphosis or to a specific signaling role that these metabolites can have through development.

In addition to its role as antioxidant, ovothiols have also been reported to have antiproliferative and anti-inflammatory activities [2,10]. Our results provide the first evidence that OvoA is present in copepods, decapods, and amphipods, opening new questions on its distribution also among other zooplanktonic species. The high expression of OvoA in *C. finmarchicus* in response to a toxic diet as well as during the key transition stages of the molt cycle, might suggest that ovothiol A has an antioxidant role also in copepods. In the recent years, drug discovery has focused on marine planktonic organisms, rather than macroorganisms because of their advantage of easy culturing in closed controlled systems and to obtain huge biomass [36,37]. Both phytoplanktonic and zooplanktonic species, have been shown to have the capability of producing several antioxidant, anti-inflammatory, anti-diabetes, anticancer, and other bioactivities compounds useful for the prevention and treatment of human pathologies [38,39,40,41,42]. Thus, the findings that ovothiol A might be produced by zooplankters can have interesting possible future biotechnological applications and can stimulate further studies on planktonic metabolites for pharmaceutical, nutraceutical, and cosmeceutical applications.

## 4. Materials and Methods

### 4.1. In Silico Mining, Reciprocal Blast, and Protein Structural Analysis

In silico search for putative OvoA encoding transcript was performed using a well-established vetting protocol that involves a mining, a reciprocal BLAST, and a structural motif analysis [43,44]. The transcriptome shotgun assembly (TSA) database on the National Center for Biotechnology Information (NCBI) was searched (October 2021) using the OvoA sequence from *Paracentrotus lividus* (AMM72581) as query (tblastn algorithm) limiting the results to Arthropoda (taxid: 6656). Among the hits, we focused on the marine organisms, mostly represented by crustacean including the Copepoda and Malacrostacea subphyla. For completeness, we also mined the Crustybase [45] to search additional available transcriptomes for other marine arthropods. Using *P. lividus* OvoA protein sequence we mined (tblastn algorithm) transcriptomes for the decapods *Eriocheir sinensis*, *Litopenaeus vannamei*, *Callinectes sapidus*, *Gecarcinus lateralis*, *Homarus americanus* whose references were not available on NCBI (Table S1). The transcripts encoding sequences OvoA for all the newly identified arthropods are provided in Supplementary File S3.

Reciprocal blast was used to confirm the identity of the putative OvoA transcripts by blasting each transcript against the non-redundant (nr) protein database. Briefly, each putative OvoA transcript was fully translated using ExPASy [46] and then the deduced protein was used to query the NCBI non-redundant (nr) protein database (blastp algorithm). Protein sequences were further inspected by searching the Pfam database for structural domains [47]. Specifically, we searched for the three expected conserved domains: the DinB-like domain, the FGE-sulfatase domain, and the methyltransferase 11 domain pertaining to the SAM-dependent methyl-transferase homologous superfamily. OvoA sequences from the calanoid *C. finmarchicus* and *C. helgolandicus* were aligned using ClustalW software (Galaxy version 2.1 [48]).

### 4.2. Phylogenetic Analysis

Phylogenetic analysis was used to support the assignment of the predicted OvoA sequences from marine arthropods, to establish their relationship to each other and to those from other metazoans. An unrooted phylogenetic tree was generated using OvoA amino acid sequences deduced from this study and from known marine metazoans [1]. The selected sequences included organisms from Mollusca, Chordata, Hemichordata, Echinodermata, Placozoa, and Porifera phylum (Table S2). An unrooted phylogenetic tree was generated using amino acid sequences that were aligned using ClustalW software (Galaxy version 2.1 [48]). FASTTREE was used to build a maximum-likelihood phylogenetic tree (Galaxy Version 2.1.10+ galaxy1) using the protein evolution model JTT+ CAT [49].

### 4.3. Expression of OvoA in Calanus finmarchicus and C. helgolandicus

Expression of OvoA transcript was examined in the copepods *Calanus finmarchicus* and *Calanus helgolandicus* exposed to stress conditions and in *C. finmarchicus* across development using previously published data [28,29]. The datasets were searched for the OvoA sequences identified in this study for *C. finmarchicus* and *C. helgolandicus*. The expression data for OvoA obtained from the three datasets was normalized using the Reads Per Kilobase per Million mapped reads RPKM method. A 2-way ANOVA (*p* < 0.05) followed by post-hoc Tukey’s test which was used to assess statistical significance in each study. A brief description of the datasets mined for the expression data is presented below.

The *C. finmarchicus* RNA-Seq dataset included expression data for females incubated for two and five days under three experimental diets: control (*Rhodomonas* sp.) and two doses (low and high) of the toxic dinoflagellate *Alexandrium fundyense* [24,50]. The toxic concentrations used for the dinoflagellate (LD = 50,000 cells L^−1^; HD = 200,000 cells L^−1^) were comparable with low and high bloom conditions reported in the Gulf of Maine [28]. Detailed methods for copepod collection, algal-incubation, RNA extraction and RNA-Seq processing are described in Roncalli et al. [28]. Briefly, females were exposed to the three diets for a total of 7 days with samples being harvested for RNA-Seq at day 2 and day 5 (three replicates/treatment). Expression was quantified by mapping each RNA-Seq library against the *C. finmarchicus* reference transcriptome (NCBI: PRJNA236528) using bowtie software (v.2.0.6). The second *C. finmarchicus* RNA-Seq dataset included expression data for six developmental stages: embryos, early nauplii (NII-NIII), early copepodites (CI), late copepodites (CIV), pre-adults (CV), and females (F) [50,51,52]. For each stage three samples were processed for RNA-Seq (exception CI and CIV with two replicates) and expression rate was measured by mapping each RNA-Seq library against the *C. finmarchicus* reference transcriptome (NCBI: PRJNA236528) using bowtie software (v.2.0.6).

Lastly, the *C. helgolandicus* dataset consisted of RNA-Seq data for laboratory-incubated females feeding for five days on the oxylipin-producing diatom *Skeletonema marinoi* and the control diet *Prorocentrum minimum*. Detailed methods for copepod collection, algal-incubation experiments, transcriptome sequencing, de novo assembly and annotation, are described in [29]. In brief, *C. helgolandicus* females collected in the Gulf of Naples (Mediterranean Sea) were fed for five days with either *S. marinoi* or *P. minimum* at 1 mg C L^−1^ (three replicates each). RNA-Seq libraries were pooled to generate a de novo assembly (NCBI: PRJNA640515) that was used to quantify expression levels by self-mapping using bowtie. Reads were normalized by length using the RPKM methods Reads Per Kilobase per Million mapped reads (RPKM).

## 5. Conclusions

This study reports for the first time the presence of the OvoA gene, a key player of the ovothiol biosynthetic pathway, in arthropods. By mining the new transcriptomic resources for marine arthropods, we report a single OvoA gene in copepods, decapods, and amphipods. Phylogenetic analysis places all the marine arthropod sequences in two separate branches, suggesting possible events through evolution. Changes in expression of the OvoA gene across development and under stress conditions, suggest that ovothiol may play a role as a defensive compound in *C. finmarchicus*, thus proposing this gene as a new biomarker of stress in holozooplanktonic species. The finding that many copepods have the OvoA gene and are thus capable of producing bioactive compounds opens up further possibilities for both new drug discovery as well as in the marine biotechnology field.

## Figures and Tables

**Table 1:** OvoA in marine arthropods. The list includes the organisms for which a single OvoA transcript was found as result of the in silico transcriptome mining that included a reciprocal blast and structural domain analysis. For each organism, phylum, subphylum, subclass, and order are listed. For each sequence, detailed information on the mined database and National Center for Biotechnology Information (NCBI) accession number are provided in Table S1.

Phylum	Subphylum	Subclass	Order	Organism
Arthropoda	Crustacea	Copepoda	Calanoida	*Neocalanus flemingeri*
				*Calanus finmarchicus*
				*Calanus helgolandicus*
				*Labidocera madurae*
				*Eurytemora affinis*
				*Temora longicornis*
				*Pseudodiaptomus annandalei*
				*Rhincalanus gigas*
				*Pleuromamma xiphias*
				*Hemidiaptomus amblyodon*
				*Metridia pacifica*
			Cyclopoida	*Eucyclops serrulatus*
				*Apocyclops royi*
				*Paracyclopina nana*
			Harpacticoida	*Tigriopus californicus*
				*Tigriopus japonicus*
				*Tisbe furcata*
			Siphonostomatoida	*Caligus rogercresseyi*
				*Lepeophtheirus salmonis*
		Malacostraca	Amphipoda	*Gammarus fossarum*
				*Gammarus pulex*
			Decapoda	*Paralithodes camtschaticus*
				*Halocaridinides trigonophthalma*
				*Scylla paramamosain*
				*Eriocheir sinensis*
				*Callinectes sapidus*
				*Gecarcinus lateralis*
				*Homarus americanus*

## Data Availability

Bioproject and transcriptome shotgun assembly (TSA) numbers of the National Center for Biotechnology Information (NCBI) database for the datasets examined in the present study are indicated in Supplementary Table S1. In Supplementary File S3, the OvoA transcript encoding protein identified in this study for marine arthropods is indicated.

## Supplementary Materials

The following are available online at https://www.mdpi.com/article/10.3390/md19110647/s1, Table S1: List of in publicly available marine arthropod datasets mined for OvoA using the query from *P. lividus*. For each dataset, information on Genus, Species, Phylum, class, and Order are provided. Accession number of the mined transcriptome (TSA database) and the Bioproject are listed. For *Eurytemora affinis* and *Tigriopus californicus* the protein sequence database was mined (blastp algoritm). For *Lepeophtheirus salmonis* the TSA accession number was not provided. For three decapods, transcriptomes were mined using CrustyBase repository. Table S2: List of OvoA protein sequences used for the phylogenetic analysis. The list includes sequences identified in this study (light blue) and sequences publicly available for selected marine metazoan [1]. For each sequence, information on Genus, Species, Phylum, class, and Order are provided. Supplementary File S3: FASTA file of OvoA transcript encoding protein identified in this study for marine arthropods.
